# A Deep Adversarial Approach Based on Multi-Sensor Fusion for Semi-Supervised Remaining Useful Life Prognostics

**DOI:** 10.3390/s20010176

**Published:** 2019-12-27

**Authors:** David Verstraete, Enrique Droguett, Mohammad Modarres

**Affiliations:** 1 Center for Risk and Reliability, University of Maryland, College Park, MD 20742, USAmodarres@umd.edu (M.M.); 2 Department of Mechanical Engineering, University of Chile, Santiago 8320000, Chile

**Keywords:** generative adversarial networks, variational autoencoders, prognostics and health management, remaining useful life, multi-sensor fusion

## Abstract

Multi-sensor systems are proliferating in the asset management industry. Industry 4.0, combined with the Internet of Things (IoT), has ushered in the requirements of prognostics and health management systems to predict the system’s reliability and assess maintenance decisions. State of the art systems now generate big machinery data and require multi-sensor fusion for integrated remaining useful life prognostic capabilities. When dealing with these data sets, traditional prediction methods are not equipped to handle the multiple sensor signals in unison. To address this challenge, this paper proposes a new, deep, adversarial approach to any remaining useful life prediction in which a novel, non-Markovian, variational, inference-based model, incorporating an adversarial methodology, is derived. To evaluate the proposed approach, two public multi-sensor data sets are used for the remaining useful life prediction applications: (1) CMAPSS turbofan engine dataset, and (2) FEMTO Pronostia rolling element bearing data set. The proposed approach obtains favorable results when against similar deep learning models.

## 1. Introduction

Reliability is defined as the ability of a product or system to perform its required functions without failure for a specified time, and when used under specified conditions. Therefore, reliability engineering has long been tasked with predicting the remaining useful life of systems by incorporating all available data. Reliability engineering has been given technologies incorporating cheap sensing with the Internet of Things (IoT) generating multi-dimensional data sets through Industry 4.0 [1]. With this new data at the engineer’s fingertips, more sophisticated methodologies to handle this data have been developed and expanded within the prognostics and health management (PHM) field. 

These data sets are often costly and time-consuming to label [2]. The engineer therefore must make an economic decision on how much data to label. Therefore, the greatest economic benefit would be to take advantage of unsupervised learning-based methodologies. To understand relevant system health states without labeling, deep learning methodologies have been shown to be a technique employed without the need for previous knowledge of degradation processes [3]. 

Most recently, remaining useful life (RUL) research focused on fully supervised deep learning methodologies has had success in RUL prediction [4,5,6,7,8,9,10,11,12,13,14,15,16,17,18,19]. These models depend on the analyst having access to a fully labeled dataset. Therefore, these RUL prediction accuracies require the use of accurate training data labels. Moreover, this previous research does not attempt to develop the underlying generative or inference model. 

A reliability engineer does not always have the resources to label all the data necessary to train a deep learning model. A valuable methodology would provide the flexibility to include a small percentage of labeled data as it becomes available and as resources allow. Generative modeling is a class of modeling techniques which provides the ability to predict RUL without having to label what could be massive multidimensional sensor data.

There have been recent efforts in generative modeling research, although it has yet to be adapted and applied to reliability and machine health prognostics. Indeed, Bayer and Osendorfer [20] and Chung et al. [21] both employed the variational autoencoder (VAE) principles to times series observations. Krishnan et al. [22] encodes the state space assumptions from within their proposed structure inference deep Kalman filter-based methodology. Karl et al. [23] proposes a VAE principled state-space filtering methodology in which the latent space is forced to fit the transition. Mescheder et al. [24] present VAEs based on adversarial training, and they achieve the flexibility to represent families of conditional distributions over latent variables. Hu et al. [25] combine generative adversarial networks (GANs) with VAE by proposing a new interpretation of adversarial domain adaptation (ADA) and a unifying generative modeling framework named through comparisons with the wake–sleep algorithm [26]. These methods, while suited for their applications in computer science, lack the requirements for RUL predictions, such as time series applications. The Markovian assumption is also utilized, where it is assumed that all information of past observations is contained within the last system state; however, for PHM, this is insufficient. Multiple operating conditions increase the degradation complexity of the RUL predictions, and some degradation paths are inherently non-Markovian (e.g., crack growth). VAE on their struggle with low probability events, like curb strike events, inherent in large systems [27]. Additionally, for PHM applications with unsupervised RUL, these methods lack the VAE combined with the adversarial training of a GAN on time-series data to provide predictions. 

To address these problems, this paper proposes a deep generative state-space modeling methodology for the remaining useful life prognostics of physical assets. The mathematical framework underpinning the proposed methodology delivers the following novel contributions for RUL predictions: (i) Non-Markovian transitions from multi-dimensional sensor data by generalizing a deep generative filtering approach for remaining useful life estimation of the system; (ii) a modeling approach that incorporates both variational and adversarial mechanisms; (iii) flexibility to handle both unsupervised and semi-supervised learning for the estimation of the remaining useful life. This method has vast applications for RUL predictions on both new and existing system assets.

The rest of the paper is organized as follows. Section 2 provides a brief overview of GAN, VAE and state-space modeling. Section 3 presents the proposed methodology and the underlying mathematical framework. Section 4 overviews the experimental results. Section 5 concludes with discussions and future work.

## 2. Background

The generative modeling research as mentioned above [20,21,22,23,24,25], all aims to tackle the problem of both a generative manifold space and inference modeling for prediction. There are slight differences between generative and inference modeling, but fundamentally they aim to solve the same problem: black-box neural transformations for implicit distribution modeling between the latent and visible spaces. For RUL estimation, reliability and PHM, this is equivalent to modeling the underlying degradation space, *z*, that is a result of the acquired observed sensor data set, *x*.

**Figure 1:**
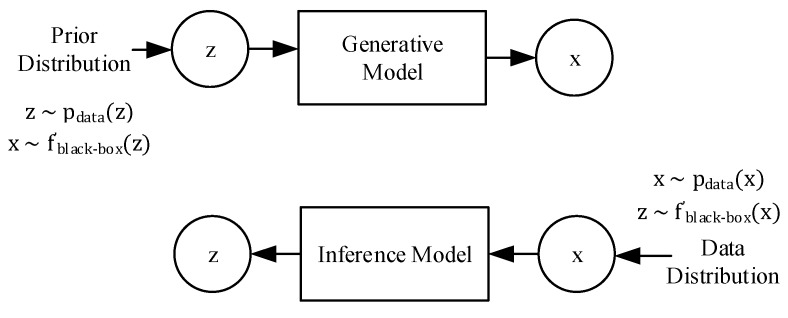
Generative and inference modeling similarities.

Traditional generative modeling approaches tend to distinguish between latent and visible variables clearly, and to treat them differently. However, a key aspect of generative modeling is that a clear boundary between the latent and visible variables (as well as generation and inference) is not necessary. Instead, viewing generative modeling as a symmetric pair helps in modeling and understanding as shown in Figure 1.

### 2.1. Generative Adversarial Networks

**Figure 2:**
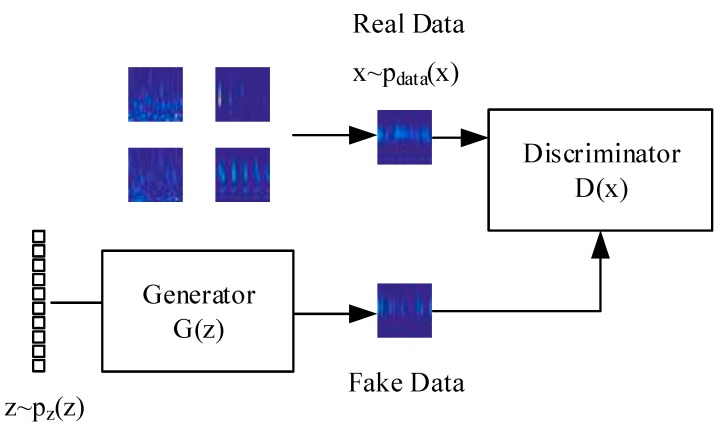
Generative Adversarial Networks (GANs).

Generative Adversarial Networks (GANs) are a class of generative modeling techniques where two neural networks compete via a minimax game [28]. This game’s objective is to develop/learn a generator distribution $P_{G}\left(x\right)$ able to generate fake data identical to the real data distribution $P_{data}\left(x\right)$. However, the generator does not directly have access to the real data. Instead, the generator distribution, $P_{G}\left(x\right)$, transforms a vector of random noise, $P_{z}\left(z\right)$, with an objective function, G(z). The generator is then trained against an adversarial discriminator network parameterized by a separate neural network whose objective, D(x), is to classify the data as real or fake, as shown in Figure 2.

There is no mechanism within the GAN training to constrain and control the Nash Equilibrium point; however, the optimal discriminator $D\left(x\right)=P_{data}\left(x\right)/\left[P_{data}\left(x\right)+P_{G}\left(x\right)\right]$ should converge to equilibrium [29]. Formally, Equation (1) shows this value function:

(1) $$\underset{G}{\min}\underset{D}{\;\max\;}V\left(G,D\right)=E_{x\sim P_{data}\left(x\right)}\left[\log(D\left(x\right)\right]+E_{z\sim P_{z}\left(z\right)}\left[\log\left(1-D\left(G\left(z\right)\right)\right)\right].$$

where *G*(*z*) is the generator objective function, *D*(*x*) is the discriminator objective function, *P_data_*_(*x*)_ is the data distribution and *P_z_*_(*x*)_ is the noise distribution.

### 2.2. Variational Autoencoders

Variational autoencoders (VAEs) are a class of generative models which develop both an inference and a generative model [27]. VAEs attempt to develop a model of latent variables, z, which can generate the observed data, x. Formally, this is expressed as:

(2) $$p\left(x\right)=\int^{\;}p\left(x,z\right)dz=\int^{\;}p\left(x|z\right)p\left(z\right)dz$$

It is common for $p\left(x|z\right)\equiv p_{\theta }\left(x|z\right)$ to be developed and parameterized by a neural network with parameters θ. For most cases, the posterior distribution p(z|x) is intractable. 

However, an approximate posterior distribution, $q_{\phi }\left(z|x\right)$, can be used to maximize the evidence lower bound (ELBO) on the marginal data log-likelihood:

(3) $$logp\left(x\right)\geq \underset{q_{\phi }\left(z|x\right)}{E}\left[logp_{\theta }\left(x|z\right)\right]-KL\left(q_{\phi }(z|x\right)||p\left(z\right))]$$

**Figure 3:**
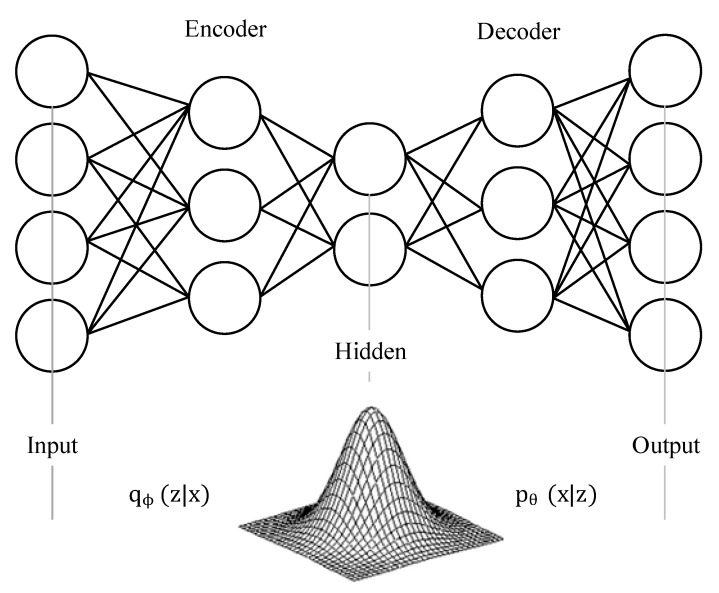
Variational autoencoder.

From this, the VAE objective is equivalent to minimizing the Kullback–Liebler (KL) divergence between $q_{\phi }\left(z|x\right)$ and $p_{\theta }\left(x|z\right)$, where $p_{\theta }\left(x|z\right)$ and $q_{\phi }\left(z|x\right)$ are parameterized by two neural networks with parameters ϕ and *θ*, as shown in Figure 3.

The training of a VAE involves the training of two neural networks, the encoder, $q_{\phi }\left(z|x\right)$ sometimes referred to as the recognition model, and the decoder, $p_{\theta }\left(z|x\right)$ sometimes referred to as the generative model. The encoder learns the relevant features of the input data and compresses the information to the latent hidden space. The decoder then attempts to generate signals (e.g., images) identical to the input data, and the reconstruction error is then minimized. 

Within the computer vision community, VAEs tend to produce blurred images that are not as sharp as those produced by other generative models. Within an engineering context, VAEs on their own can result in a common issue with particle filtering algorithms: Without a fully expressive generative model capable of handling extremely low probability events or sensor reading interactions, the resulting prognosis model may not have considered these non-Markovian events.

## 3. Proposed Methodology

**Figure 4:**
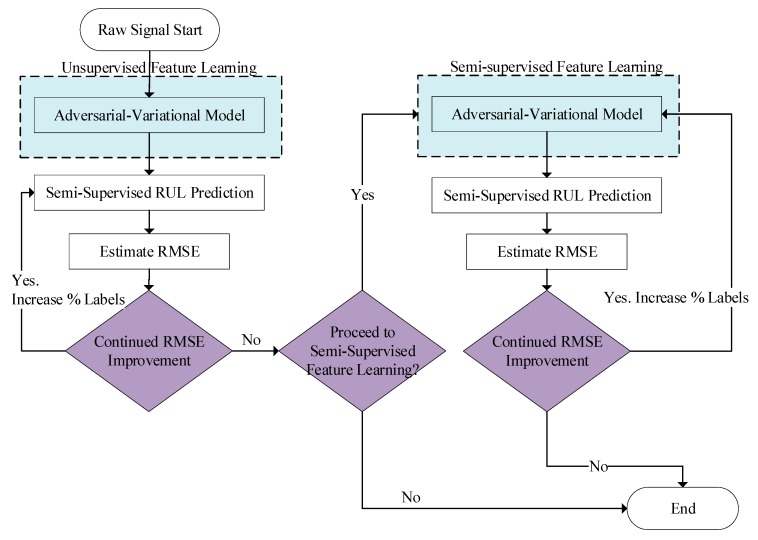
Proposed deep generative methodology for remaining useful life estimation.

Given the complexities and associated uncertainty of the fault diagnostic and prognostic problem, a proposed methodology would be one that is flexible enough to include new sets of information as they become available. Expert opinion, black swan events, abnormal operating conditions, knowledge of the underlying failure modes, physics of failure models and partially relevant information, can all be included within the remaining useful life estimation. While this information can be valuable, the methodology should also adequately generalize this data. For example, extracting relevant features, which may be known, may not be able to account for noisy sensor signals or operating conditions outside the norm. With this end, we propose the methodology shown in Figure 4.

The methodology has two distinct phases: (1) Unsupervised learning assessment of RUL, (2) Semi-supervised learning assessment of RUL. It starts with the raw data signal fed into the unsupervised variational adversarial filter. Without knowledge of labeling (e.g., the system health states) at the start of operation of the system, this stage of development requires the use of unsupervised remaining useful life estimation. Once the system has had operational time, the engineer can start labeling data in a semi-supervised iterative loop, i.e., to identify the system’s health states with corresponding input sensor data patterns. As it may not be feasible (time and cost-wise) to do so for all the available data, experiments have shown that semi-supervised methodologies with only a few percentages of the data set labeled can substantially improve the unsupervised methods [30]. Therefore, as the engineer labels data, the framework is robust enough to handle this percentage of labeled data, as shall be demonstrated later in Section 4. 

### 3.1. Unsupervised Remaining Useful Life Formulation

**Figure 5:**
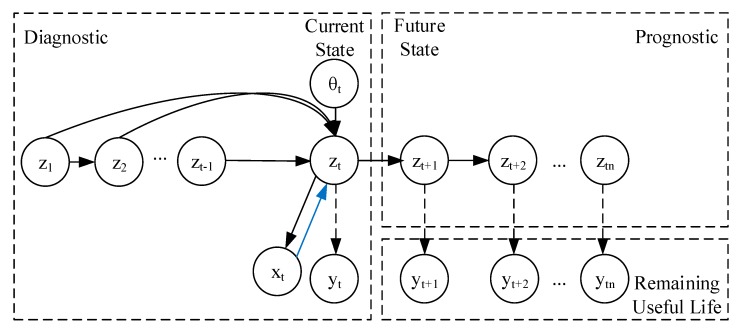
Forward graphical model for the proposed mathematical framework.

**Figure 6:**
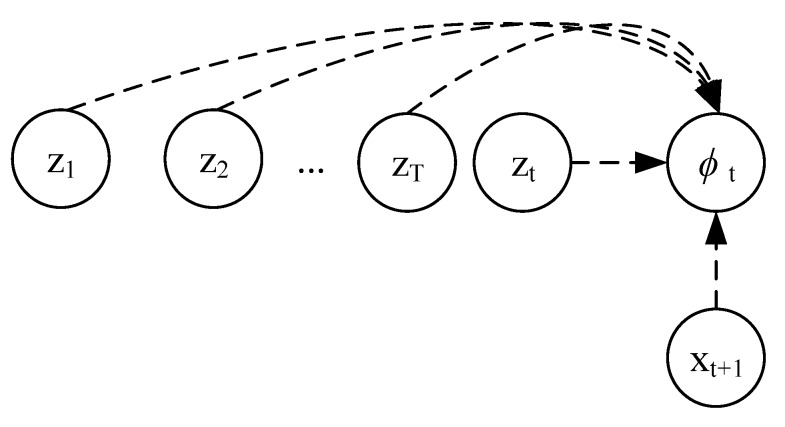
Inference training model.

In this work, we propose a mathematical formulation that encapsulates the following features: Both unsupervised and semi-supervised feature learning, adversarial-variational state-space modeling with non-Markovian transitions (i.e., it is not assumed that all information regarding past observation is contained within the last system state), adversarial training mechanism on the training of the recognition $q_{\phi }\left(z_{t}|x_{1:t}\right),$ and variational Bayes for the inference and generative model $p_{\theta }\left(x_{t}|z_{1:t}\right)$. As shown in Figure 5 and Figure 6, we set $x_{t}$ as the observed sensor data, $z_{t}$ as the latent system health state (e.g., crack length, degradation), and $y_{t}$ is the target domain relevant to the adversarial training y ∈0,1,…,RUL. Blue lines represent the adversarial mechanism, dashed lines indicate inference processes and solid lines indicate a generative process. The transition parameters, $\theta_{t}$, are inferred via a neural network. Past observations are directly included in the inferential model output. The proposed mathematical framework does not assume that all the information relevant to parameters $\phi_{t}$ is encoded within $z_{t}$.

To establish the training optimization, we denote the latent sequence $z_{t}\in Z\subset {\mathbb{R}}^{n_{z}}$ as a set of real numbers $n_{z}$ and observations as $x_{t}\in X\subset {\mathbb{R}}^{n_{x}}$. Now X can be, but is not limited to, a multi-dimensional sensor data set from a large asset. The observations, $x_{t}$, are not constrained to a Markovian transition assumption. For engineering problems (e.g., crack growth and environmental effects on RUL) these transitions can be complex non-Markovian. Therefore, the degradation sequence $p\left(x_{t}|z_{1:t-1}\right)$ generated by the discrete multi-dimensional sensor data sequences $x_{t}=\left(x_{1},x_{2},\ldots ,x_{t}\right)$ and latent sequences $z_{1:t-1}=\left(z_{1},z_{2},\ldots ,z_{t-1}\right)$ are of interest to the engineer. This is shown in Equation (4):

(4) $$p\left(x_{t}|x_{1:t-1}\right)=\int^{\;}p\left(x_{t}|x_{1:t-1},z_{1:t}\right)p\left(z_{1:t}|z_{1:t-1}\right)dz_{1:t}$$

where $z_{1:t-1},z_{t}\in Z\subset {\mathbb{R}}^{n_{z}}$ denotes the latent sequence. The basis of the latent dynamical system is assumed to have an emission model $p\left(x_{t}|x_{1:t-1},z_{1:t}\right)$ and transition model $p\left(z_{t}|z_{1:t-1}\right)$. Two assumptions are classically imposed on the emission and transition models as shown in Equations (5) and (6),

(5) $$p\left(x_{t}|x_{1:t-1},z_{1:t}\right)=\overset{t}{\underset{i=1}{\prod }}p\left(x_{t}|z_{t}\right)$$

(6) $$p\left(z_{t}|z_{1:t-1}\right)=\overset{t-1}{\underset{i=0}{\prod }}p\left(z_{t+1}|z_{t}\right)$$

These equations capture the assumption that the current state, $z_{t}$, holds complete information for the observations $x_{t}$, and the subsequent state $z_{t+1}$. For noisy multidimensional sensor data sets with complex non-Markovian transition, this assumption is insufficient. The proposed mathematical formulation characterizes the state-space model without these assumptions.

Therefore, to derive the proposed mathematical framework of the proposed methodology, we first put forward the variational lower bound objective function from Equation (4), given that we do not make the Markov assumption from Equations (5) and (6). Thus, we have:

(7) $$KL\left(q_{\phi }\left(z_{1:t}|x_{1:t}\;\right)||p\left(z_{1:t}|x_{1:t}\;\right)\right)=-\int^{\;}q_{\phi }\left(z_{1:t}|x_{1:t}\;\right)\left[log\left(\frac{p\left(z_{1:t}|x_{1:t}\;\right)}{q_{\phi }\left(z_{1:t}|x_{1:t}\;\right)}\right)\right]$$

As we know,

(8) $$p\left(z_{1:t}|x_{1:t}\right)=\frac{p(x_{1:t},z_{1:t})}{p\left(x_{1:t}\right)}$$

Substituting into Equation (7) we get,

(9) $$=-\int^{\;}q_{\phi }\left(z_{1:t}|x_{1:t}\right)\left[log\left(\frac{\frac{p(x_{1:t},z_{1:t})}{p\left(x_{1:t}\right)}}{q_{\phi }\left(z_{1:t}|x_{1:t}\right)}\right)\right]$$

Rearranging (9) we get,

(10) $$=-\int^{\;}q_{\phi }\left(z_{1:t}|x_{1:t}\right)\left[log\left(\frac{p(x_{1:t},z_{1:t})}{q_{\phi }\left(z_{1:t}|x_{1:t}\right)}*\frac{1}{p\left(x_{1:t}\right)}\right)\right]$$

Applying the product rule on (10) we have,

(11) $$=-\int^{\;}q_{\phi }\left(z_{1:t}|x_{1:t}\right)\left[log\frac{p(x_{1:t},z_{1:t})}{q_{\phi }\left(z_{1:t}|x_{1:t}\right)}+log\frac{1}{p\left(x_{1:t}\right)}\right]$$

Applying the quotient rule on (11) we have,

(12) $$=-\int^{\;}q_{\phi }\left(z_{1:t}|x_{1:t}\right)\left[\log\frac{p(x_{1:t},z_{1:t})}{q_{\phi }\left(z_{1:t}|x_{1:t}\right)}-\log p\left(x_{1:t}\right)\right]$$

Separating (12) we have,

(13) $$=-\int^{\;}q_{\phi }\left(z_{1:t}|x_{1:t}\right)\left[log\frac{p(x_{1:t},z_{1:t})}{q_{\phi }\left(z_{1:t}|x_{1:t}\right)}\right]+\underset{z}{\int }q_{\phi }\left(z_{1:t}|x_{1:t}\right)logp\left(x_{1:t}\right)$$

However, we know that,

(14) $$logp\left(x_{1:t}\right)\underset{z}{\int }q_{\phi }\left(z_{1:t}|x_{1:t}\right)=1$$

Therefore, we have,

(15) $$logp\left(x_{1:t}\right)=KL\left[q_{\phi }\left(z_{1:t}|x_{1:t}\;\right)||p\left(z_{1:t}|x_{1:t}\right)\right]+\int^{\;}q_{\phi }\left(z_{1:t}|x_{1:t}\right)\left[log\frac{p(x_{1:t},z_{1:t})}{q_{\phi }\left(z_{1:t}|x_{1:t}\;\right)}\right]$$

where we simultaneously want to minimize the Kullback–Liebler (KL) divergence and maximize the variational (evidence) lower bound (ELBO), $ℒ\left(\theta ,\phi ;x_{1:t}\right)$, as shown in Equation (16):

(16) $$ℒ\left(\theta ,\phi ;x_{1:t}\right)=\int^{\;}q_{\phi }\left(z_{1:t}|x_{1:t}\;\right)\left[log\frac{p(x_{1:t},z_{1:t})}{q_{\phi }\left(z_{1:t}|x_{1:t}\right)}\right]$$

Now, rearranging Equation (16), we have the non-Markovian variational lower bound derived for time series data in Equation (17):

(17) $$ℒ\left(\theta ,\phi ;x_{1:t}\right)=E_{q_{\phi }\left(z_{1:t}|x_{1:t}\;\right)}\left[logp_{\theta }\left(x_{1:t}\;|z_{1:t}\right)\right]-KL\left[q_{\phi }\left(z_{1:t}|x_{1:t}\right)p(z_{1:t})\right]$$

To add in adversarial training, we follow Goodfellow, et al. [28] and rewrite the optimization function from Equation (17) to Equation (18) as follows:

(18) $$\underset{\theta }{min}\underset{\phi }{\;max}E_{D(x)}E_{q_{\phi }\left(z_{1:t}|x_{1:t}\;\right)}\left(\left[logp_{\theta }\left(x_{1:t}|z_{1:t}\right)\right]-KL\left[q_{\phi }\left(z_{1:t}|x_{1:t}\right)p(z_{1:t})\right]\right)$$

We now have an objective function which gives us an expressive $q_{\phi }\left(z_{t}|x_{t},z_{1:t-1}\right)$, that is, we have a mathematical framework the characterizes the state-space model without the restrictive assumptions outlined in Equations (5) and (6). Additionally, this mathematical framework contains both the generative and inference models of the system state that allows us to perform fault diagnostics and prognostics as well as the RUL of the system assessment.

### 3.2. Semi-Supervised Loss Function

Semi-supervised initialization involves training of the chosen model’s architecture with an incrementally increasing set of labeled data. This is an important aspect to explore, because as the engineer gains more knowledge about a new system, one can label small sets of data, which are known to be system degradation versus healthy operation to increase the system’s health state prediction [29]. This approach can improve the quality of the results via a semi-supervised loss, L, function given by Equation (19):

(19) $$L=L_{supervised}+L_{unsupervised}$$

In the context of the proposed adversarial framework, during the unsupervised training, the discriminator learns features to avoid classifying the generated data as real data, but these features might not be the best representation. To improve the discriminator and develop more meaningful features for the system’s health states over time, labels are used. This is possible by writing the loss function, L, within training to some predetermined number of epochs as follows:

(20) $$L_{supervised}=-E_{x_{1:t},y_{1:t}\sim p_{data}\left(x_{1:t},y_{1:t}\right)}logp_{model}(y_{1:t}|x_{1:t},y_{1:t}<K+1)$$

(21) $$\begin{array}{c} L_{unsupervised}=-\{E_{x\sim p_{data}\left(x_{1:t}\right)}log[1-p_{model}(y_{1:t}\\ \;=K+1|x_{1:t})]+E_{x\sim G}log[p_{model}(y_{1:t}=K+1|x_{1:t})]\} \end{array}$$

where *x* and *y* are the same as defined previously. *p_model_* corresponds to the trained model. This cost function adds a cross-entropy loss for the first K discriminator outputs. The unsupervised cost is the same as the original GAN (see Equation (2)). However, there is a slight change, as now K + 1 corresponds to the probability of the sample being false [31]. The discriminator is used as a competent classifier, given a subset of the dataset. In the context of the proposed mathematical framework, the discriminator will be used as a feature extractor, given a subset of the dataset to improve the system’s health state identification results.

## 4. Results and Discussion

The Commercial Modular Aero-Propulsion System Simulation (C-MAPSS) multi-sensor data set was used to evaluate the proposed methodology [32]. C-MAPSS is a simulation tool developed in MATLAB and Simulink environment for commercial turbofan engines. The model outputs multiple sensor signal values corresponding to the input parameters of an engine component degradation level or health indicator. In order to adjust to a specific problem that the user is trying to solve, operational profile, closed-loop controllers and environmental conditions, can all be adjusted. The term “multi-sensor” for this data set references multiple types of sensors, operating conditions, environmental conditions, flight numbers and trajectories. The 90,000-pound thrust class engine and the CMAPSS simulation package allows operations at (1) Mach numbers from 0 to 0.90, (2) Altitudes measuring sea level to 40,000 feet, and (3) sea-level temperatures measuring −60 to 103 °F.

**Figure 7:**
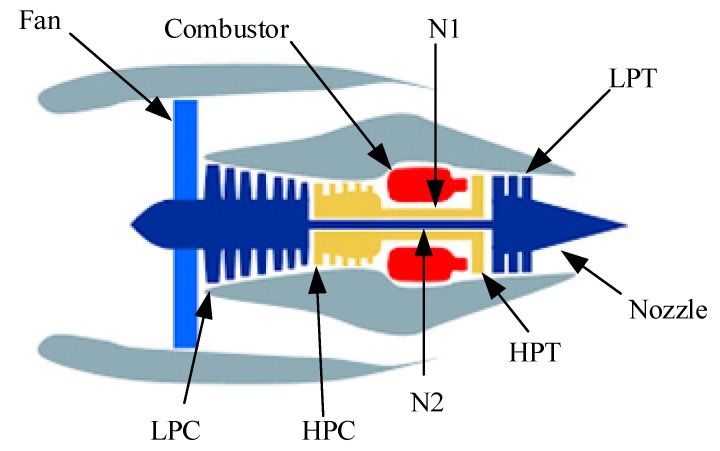
Simplified diagram of engine simulated in the Commercial Modular Aero-Propulsion System Simulation (C-MAPSS) [32].

Figure 7 shows the engine’s main elements with the following abbreviations: fan speed (N1), low-pressure turbine (LPT), low-pressure compressor (LPC), high-pressure compressor (HPC), core speed (N2), high-pressure turbine (HPT).

The PHM 2008 competition data set developed from using the C-MAPSS program is used as an example of application in this paper [33]. Four data sets, FD001 through FD004, are available, and have the properties shown in Table 1

**Figure 8:**
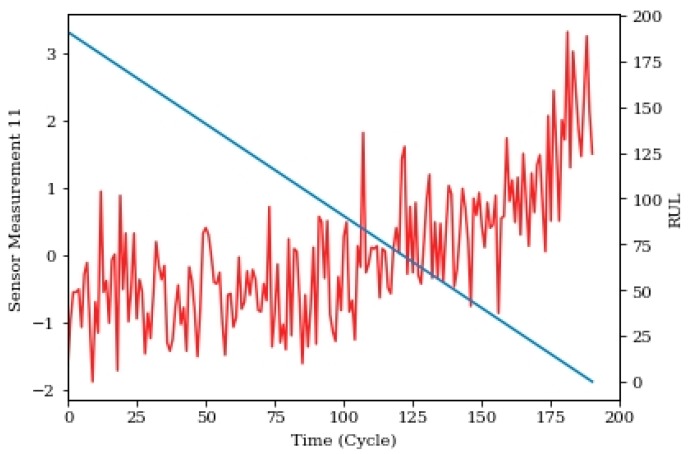
CMAPSS Sensor measurement 11 and remaining useful life (RUL) versus cycle.

The four data sets have a combination of two fault conditions: high-pressure compressor (HPC) degradation and fan degradation. The data set includes the true RUL to measure prediction performance against. The data set is separated into training and test sets consisting of 26 different sensor measurements, three conditions of operation, flights and mission times. Each of the engines within the dataset initiates with different levels of manufacturing variation and initial degradation. This information is hidden from the engineer, and is not considered a fault condition. The three operational settings do have a substantial effect upon engine performance. These settings are known. Finally, the sensor data is contaminated with noise. Figure 8 shows an example of one of the sensor measurements for one trajectory with its RUL.

To avoid unnecessary duplications, the following sections only use FD001 and FD004 for the sake of brevity. To successfully predict the remaining useful life of the engine, a methodology that can fuse the twenty-six different sensor signals is necessary.

### 4.1. Example of Application: Turbofan Engines

To evaluate the proposed semi-supervised methodology, two types of labeling were used: (1) fixed interval and (2) random interval. The fixed interval consists of labeling one out of every *x* number of labels (i.e., 5% equals labeling 1 out of every 20 data points.) Random interval labeling consisted of taking a random sample of the complete data set for labeling (i.e., 5% of 15,680 data points equals 784 randomly labeled data points). This was done because, as the time interval between labels is decreasing, the RUL estimation error improvements reduce. As one will notice in the rest of this section, this did affect RUL prognostics.

To evaluate the effects of adding a small subset of labeled data to the training procedure, semi-supervised learning was also conducted on the C-MAPSS dataset. There are two parts of the algorithm to evaluate this effect of labeling on the results: (1) feature learning and (2) regression. When it is stated “semi-supervised feature learning”, it implies that the percentage of labels were fed into the feature learning phase of the model. When results are reported as “unsupervised feature learning”, zero labels were used in the feature learning portion of the model.

The proposed methodology is evaluated against the true RUL via the root mean square error (RMSE). To not sway these results in a more positive light, the authors chose to train the model ten times and take the average results from all ten.

**Figure 9:**
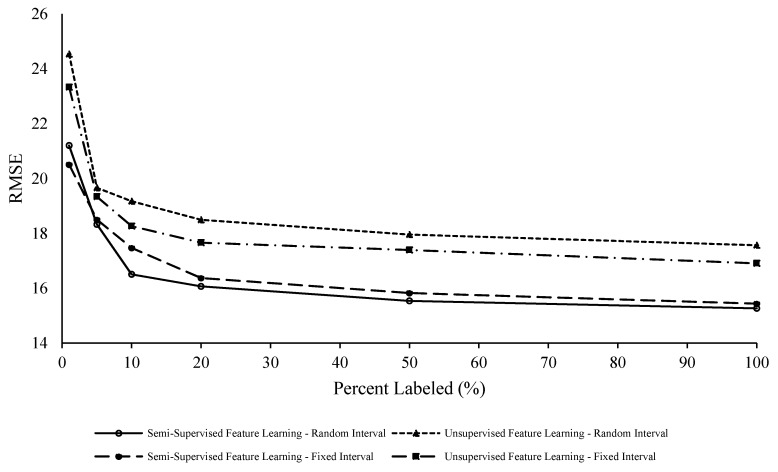
FD001 RMSE versus percent labeled (%).

First, FD001 is evaluated from one percent to one hundred percent labels. The RMSE results can be found in Table 2 and Table 3. As one can see from the results, there is an effect on the RUL prognostics with both types of labeling (fixed vs. random) and adding labels to both parts of the model. This can also be viewed in Figure 9 There are two observations to note when looking at the results: (1) adding labels to feature learning improves the RUL prediction, and (2) as more labels are added to the feature learning and regression parts of the modeling, the prediction performance (in terms of RMSE) improvement tends to taper off after twenty percent. The increase in prediction performance from adding labels to the feature learning portion of the model shows that feeding labels to the generative model help extract more degradation-related features present in the data. The appropriate percentage of labeling could be inferred or determined based on the evolution of the RMSE according to Figure 9. In this case, the RMSE marginally improves for FD001 beyond twenty percent labeling (1.5% improvement for 50% labeling and 2.7% improvement for 100% labeling). This is important because labeling data is expensive and time consuming. Therefore, increasing the prediction performance (i.e., reducing RMSE) beyond twenty percent of labels becomes increasingly more expensive for a smaller benefit.

**Figure 10:**
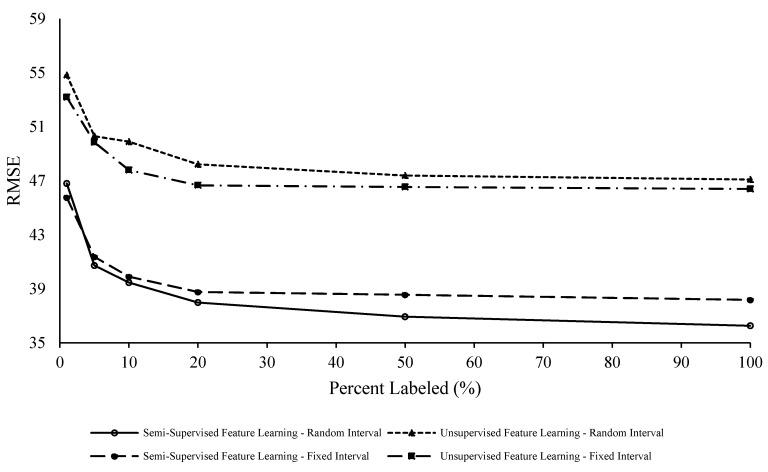
FD004 RMSE versus percent labeled (%).

To evaluate the effects and differences of modeling operating conditions and additional fault modes, FD004 was also examined. This data set is more applicable for cases that include fleets of vehicles operating in different conditions. Based on the results reported in Figure 10, Table 4 and Table 5, this data set had a larger improvement in results by adding labels into both feature learning and regression parts of the model. One can argue that this is because of the non-homogeneity of the data resulting from the inclusion of additional operating conditions and fault modes.

Note that FD004 needed an increased percentage of labels given the inherent non-homogeneity of the data set. With six operating conditions and two failure modes, there is a higher degree of uncertainty, and therefore the model performance benefits from an increasing percentage of labels. Compared to the FD001 results in Figure 9, there is still a noticeable reduction of RMSE up to 100% labeling. This reflects the model taking advantage of the increased knowledge of the RUL evolution granted by the known labels during the training stage.

Moreover, both FD001 and FD004 RUL prediction benefited from random interval labeling during semi-supervised feature learning. This is can be attributed to the proposed model’s ability to better generalize the underlying generative model or lower-dimensional manifold space. The output of the proposed framework also includes a semi-supervised model that gives the engineer the ability to continuously add labels as more information about the degradation process becomes available. From a practical point of view, this is an important characteristic of the model: the engineer can weigh the economic impacts of labeling more data.

### 4.2. Ablation Study and Comparison Results

**Figure 11:**
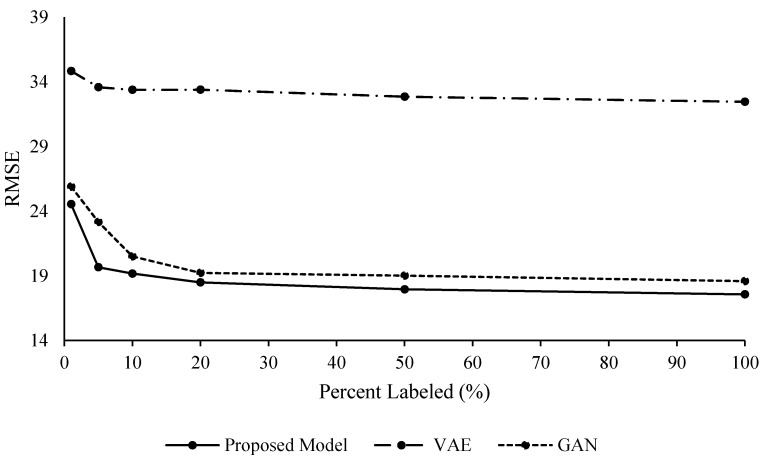
FD001 Unsupervised Feature Learning, Random Labeling Intervals.

**Figure 12:**
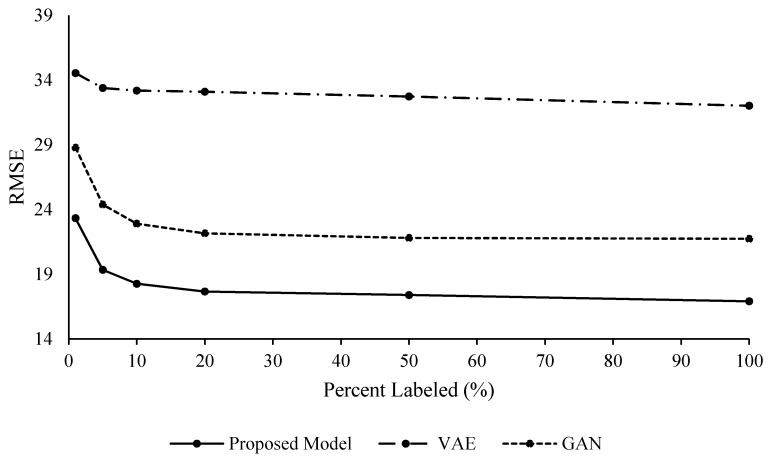
FD001 Unsupervised Feature Learning, Random Labeling Intervals.

An ablation study was conducted on the FD001 data set to understand the effects and advantages of integrating variational inference with an adversarial approach, as it is done in the proposed mathematical framework. To this end, both VAEs and GANs were applied separately to FD001 and RUL estimates were performed. Unsupervised feature learning with semi-supervised regression was performed to evaluate the effects of the generative modeling without labels for feature learning. These results can be seen in Table 6 and Table 7, Figure 11 and Figure 12.

These results allow one to see the effects of the variational and adversarial approach of the proposed methodology. Even though the VAE and GAN models provide acceptable results, the proposed methodology outperformed both on their own. The VAE model’s RUL prediction performance in terms of RMSE was slightly better with fixed interval labeling, while the GAN model’s performance was better with random intervals for the labeling. VAE did not perform as well as the GAN and the proposed methodology. The VAE model also did not benefit from labeling more data after adding 10% labels.

The authors suspect the VAE model did not perform as well due to the possibility of modeling the Gaussian priors of the VAE model sequentially in the training portion of the model [32]. These results show the value of the combination of the non-Markovian adversarial and variational capabilities within the proposed methodology.

Additionally, the proposed methodology and corresponding mathematical framework were assessed against the deep generative modeling technique outlined in Krishnan, et al. [22]. This modeling technique incorporates a Deep Markov Model (DMM) state-space system utilizing structured inference architecture without an adversarial mechanism. Additionally, Krishnan’s methodology was not developed for, or applied to, the PHM context. It is, however, a state-of-the-art deep generative modeling technique on time series data. For this paper, it was applied to the CMAPSS FD001 and FD004 data sets as a comparison method. These results can be found in Table 8.

As shown in Table 8, the proposed methodology provides superior results when compared with DMM. Additionally, the DMM is restricted to unsupervised learning, and does not provide a mechanism for semi-supervised learning and labeling. To compare the methodology to a more traditional fully supervised RUL approach, a basic neural network was also applied to the data set within this table. This gives a baseline for the engineer to make an economic decision about labeling the complete data set. This further demonstrates the benefits of the proposed methodology for RUL assessment.

The analysis presented was completed with an nVidia TitanXP GPU processor, and each set of training results took approximately fifty-five minutes to complete. Currently, it takes 1.1 s on average to feed a batch set of data through the model after training. Further research would be required and is beyond the scope of this paper; however, once training is complete it would be reasonable to envision the regression side of the proposed methodology as capable of operation within an online real time system.

### 4.3. FEMTO Dataset Results

**Figure 13:**
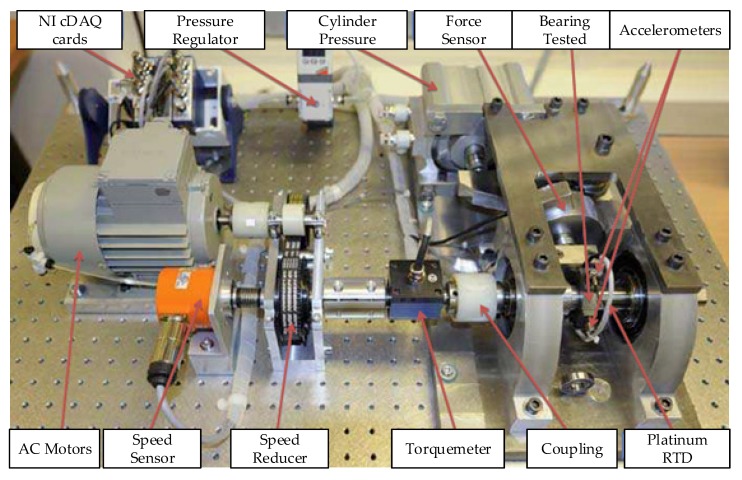
Overview of PRONOSTIA [34].

For an additional point of experimental validation, this dissertation uses the PHM 2012 Challenge dataset incorporating the PRONOSTIA platform for accelerated aging The term “multi-sensor” for this data set means multiple bearing trajectories, load conditions and rotational speeds. The experimental setup is shown in Figure 13.

The platform’s goal is to provide a sensor data output that characterizes the realistic degradation processes of rolling element bearings throughout their life. This data set consists of a run to failure data set for seventeen bearings at different load cases and rotational speeds. The information for each bearing is outlined in Table 9.

**Figure 14:**
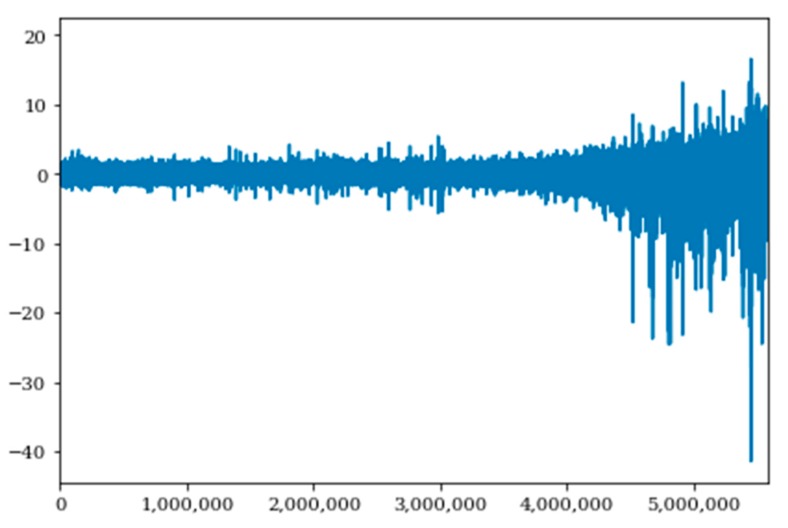
Raw Bearing 1-3 Data Signal Versus Time.

**Figure 15:**
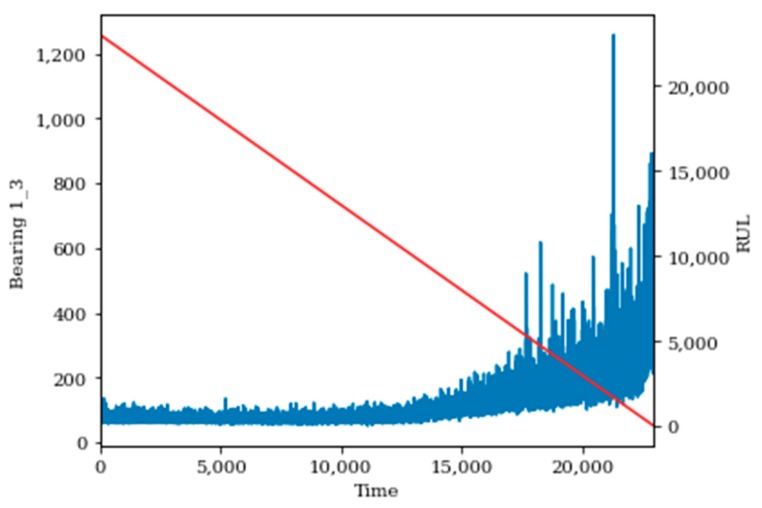
Processed Spectrogram (STFT) Degradation Data for Bearing 1–3.

To evaluate this data set, sixteen of the seventeen bearings were used as the training set, while the seventeenth bearing is used as the test set. Data augmentation in the form of spectrogram images was done to ensure a consistent signal and degradation path. Figure 14 and Figure 15 show the raw signal and spectrogram signals for bearing 1–3 prior to data normalization. Data was normalized for each bearing signal to maintain a consistent scale for the data, and it is a necessary step in preparation to be input into the proposed methodology.

The results of the FEMTO data set within the proposed methodology show good performance against research incorporating this dataset, where the published fully supervised RMSE results for bearings 1–3, 2–4 and 3–1 are 9.0, 8.9 and 24.2, respectively [35]. As shown in Table 10, the proposed methodology outperformed these results.

Similarly, to the CMAPSS results, increasing the percentage of the labels reduces the RMSE results of the prediction accuracy. This again demonstrates that the model’s ability to extract system health features improves with more knowledge about the system. Due to the nature of this data set, only fixed interval labeling was used. Random interval labeling was explored; however, unlike the CMAPSS results, it had mixed results. The results show the robust ability of the proposed methodology to generalize the underlying machine health state degradation process.

## 5. Conclusions

In this paper, a novel deep learning-enabled, adversarial–variational methodology, and corresponding non-Markovian mathematical framework, for remaining useful life estimation, was proposed. This was then applied to both a public multi-sensor turbo fan dataset and a public multi-sensor rolling element bearing data set. The proposed methodology achieved superior RUL prediction performance and demonstrated its ability to predict the RUL even with a small percentage of labeled data.

Within the ablation study, the proposed framework proved higher RUL prediction performance with a combined generative modeling methodology. The prediction performance was further enhanced with the addition of labels to the data set. Moreover, the type of labeling (random versus fixed-interval) was explored, and it was uncovered that the method with which one labels time series data can be beneficial towards RUL prediction. Preliminary results indicate that the proposed methodology could be implemented as an online system; however, further research is required to explore this possibility.

A limitation of the proposed methodology is inherent when trying to quantify the variability, while not specifically calculating the uncertainty. This is a potential drawback to this model supporting PHM risk decision making given the computational complexity. Therefore, a suggestion would be to expand the method in terms of a Bayesian framework so the uncertainty on RUL can be explicitly calculated. This approach would be useful with the implication of a quantifiable uncertainty metric, where a loss function could find a relation between the percentage of labeling and the uncertainty on RUL. From this, one could then find an optimal labeling percentage for a given physical asset’s dataset based on the risk surrounding a failed prediction.

## Figures and Tables

**Table 1:** C-MAPSS Data Overview.

Data Set	Train Trajectories	Test Trajectories	Operating Conditions	Fault Modes
FD001	100	100	1 (Sea Level)	1 (HPC)
FD002	260	259	6	1 (HPC)
FD003	100	100	1 (Sea Level)	2 (HPC, and Fan)
FD004	248	249	6	2 (HPC, and Fan)

**Table 2:** FD001 root mean square error (RMSE) Unsupervised feature learning with semi-supervised regression.

Labeling	1%	5%	10%	20%	50%	100%
Fixed	23.33	19.34	18.26	17.66	17.39	16.91
Random	24.54	19.66	19.17	18.50	17.96	17.57

**Table 3:** FD001 RMSE Semi-supervised feature learning with semi-supervised regression.

Labeling	1%	5%	10%	20%	50%	100%
Fixed	20.50	18.50	17.47	16.37	15.82	15.44
Random	21.20	18.33	16.50	16.06	15.54	15.27

**Table 4:** FD004 RMSE Unsupervised feature learning with semi-supervised regression.

Labeling	1%	5%	10%	20%	50%	100%
Fixed	53.19	49.85	47.79	46.66	46.54	46.40
Random	54.82	50.30	49.90	48.22	47.39	47.09

**Table 5:** FD004 RMSE Semi-supervised feature learning with semi-supervised regression.

Labeling	1%	5%	10%	20%	50%	100%
Fixed	45.76	41.36	39.90	38.76	38.56	38.18
Random	46.80	40.73	39.46	37.98	36.93	36.26

**Table 6:** FD001 RMSE Unsupervised Feature Learning—Fixed Labeling Intervals.

Model	1%	5%	10%	20%	50%	100%
Proposed	23.33	19.34	18.26	17.66	17.39	17.09
GAN	28.77	24.38	22.90	22.16	21.80	21.73
VAE	34.54	33.39	33.18	33.10	32.73	32.01

**Table 7:** FD001 RMSE Unsupervised Feature Learning—Random Labeling Intervals.

Model	1%	5%	10%	20%	50%	100%
Proposed	24.54	19.66	19.17	18.50	17.96	17.57
GAN	25.89	23.16	20.50	19.22	19.01	18.59
VAE	34.82	33.58	33.37	33.38	32.84	32.44

**Table 8:** Unsupervised RMSE average results for the C-MAPSS test set.

	Proposed		Krishnan		Fully Supervised NN	
Data Set	Mean	Std. Dev.	Mean	Std. Dev.	Mean	Std. Dev.
FD001	16.91	0.39	17.32	1.91	16.43	0.84
FD004	46.40	0.53	54.15	0.54	38.35	0.18

**Table 9:** FEMTO Dataset Information.

Condition	Load	Speed	Bearings			
1	4000	1800	1–1	1–2	1–3	1–4
			1–5	1–6	1–7	
2	4200	1650	2–1	2–2	2–3	2–4
			2–5	2–6	2–7	
3	5000	1500	3–1	3–2	3–3	

**Table 10:** FEMTO RMSE Results—Semi-supervised Feature Learning with Semi-Supervised regression.

Bearing	1%	5%	10%	20%	50%	100%
1–3	11.32	11.10	10.96	10.36	7.50	6.59
2–4	10.13	9.92	8.65	7.28	6.77	6.42
3–1	31.90	27.42	23.73	20.08	15.02	11.51
